# Protective and therapeutic effects of gallic acid on medication-related osteonecrosis of the jaw in rats: modulation of inflammation, fibrosis and endoplasmic reticulum/mitochondrial stress pathways

**DOI:** 10.1186/s12903-026-07732-w

**Published:** 2026-01-24

**Authors:** Tayfun Yazıcı, Mustafa Isleyen, Aybike Imeci, Halil Asci, Muhammet Yusuf Tepebasi, Abdurrahman Gülal, Oznur Kolay, Serife Tasan, Sefa Erdem Karapınar, Ozlem Ozmen

**Affiliations:** 1 Department of Oral and Maxillofacial Diseases and Surgery, Faculty of Dentistry, Suleyman Demirel University, Isparta, Turkey; 2 Department of Oral and Maxillofacial Surgery, Faculty of Dentistry, Mehmet Akif Ersoy University, Burdur, Turkey; 3 Private Dis Dostu Dental Healtcare Clinic, Isparta, Turkey; 4 Department of Pharmacology, Faculty of Medicine, Suleyman Demirel University, Isparta, Turkey; 5 Department of Medical Genetic, Faculty of Medicine, Suleyman Demirel University, Isparta, Turkey; 6 Department of Medical Pharmacology, Institute of Health Sciences, Suleyman Demirel University, Isparta, Turkey; 7 Department of Pathology, Faculty of Veterinary Medicine, Mehmet Akif Ersoy University, Burdur, Turkey; 8 Department of Orthopedics and Traumatology, Faculty of Medicine, Suleyman Demirel University, Isparta, Turkey

**Keywords:** GA, Zoledronic acid, Osteonecrosis, Mitochondrial apoptosis, Inflammation, Fibrosis

## Abstract

**Background:**

Medication-related osteonecrosis of the jaw (MRONJ) is a severe adverse effect of long-term bisphosphonate therapy characterized by impaired bone remodeling, inflammation, and fibrosis. Gallic acid (GA), a natural phenolic compound with potent antioxidant and anti-inflammatory properties, has been proposed as a protective agent against drug-induced tissue injury. This study investigated the prophylactic and therapeutic effects of GA on zoledronic acid (ZOA)–induced MRONJ in rats.

**Methods:**

Thirty-two adult female Wistar albino rats were randomly divided into four groups (*n* = 8): Control, MRONJ (ZOA + tooth extraction), MRONJ + Pre-GA (prophylactic GA), and MRONJ + Post-GA (therapeutic GA). MRONJ was induced by intraperitoneal ZOA (0.06 mg/kg/week, 5 weeks) followed by mandibular molar extraction. GA (100 mg/kg, i.p.) was administered either before or after ZOA exposure for 7 days. Mandibular tissues were analyzed histopathologically, immunohistochemically (Fibroblast growth factor [FGF], transforming growth factor beta 1 [TGF-1β], tumor necrosis factor alpha [TNF-α]), and molecularly (TNF-α, TGF-1β, endothelial nitric oxide synthase [eNOS], cytochrome c [Cyt-C], caspase-3 [Cas-3], protein kinase RNA-like ER kinase [PERK], C/EBP homologous protein [CHOP]).

**Results:**

MRONJ rats exhibited severe osteonecrosis, increased inflammation and fibrosis, disrupted collagen organization, elevated osteoclast activity, and suppressed osteoblast function. Immunohistochemistry revealed significant downregulation of FGF and TGF-1β with a concomitant rise in TNF-α. Gene expression analyses showed upregulation of TNF-α, TGF-1β, CHOP, PERK, Cyt-C, and Cas-3, along with downregulation of eNOS. GA administration markedly ameliorated these alterations, restoring bone architecture, normalizing growth factor expression, suppressing pro-inflammatory and apoptotic signaling, and reducing ER stress. The prophylactic regimen showed the most pronounced protective efficacy.

**Conclusions:**

GA confers both preventive and therapeutic benefits against MRONJ by mitigating oxidative stress, inflammation, fibrosis, ER stress and mitochondrial apoptosis. These findings suggest that GA may represent a promising adjuvant strategy for managing or preventing MRONJ in patients receiving long-term antiresorptive therapy.

## Introduction

Medication related osteonecrosis of the jaw (MRONJ) is a rare but serious complication associated with antiresorptive and anti-angiogenic therapies, principally nitrogen-containing bisphosphonate (BP)s and the Receptor activator of nuclear factor kappa-B ligand (RANKL) inhibitor denosumab [1]. In patients undergoing long-term antiresorptive treatment, particularly in oncologic settings, dental extractions or trauma often precipitate non-healing exposed bone in the jaw region, a hallmark of the disease [2]. Although the exact pathophysiology remains incompletely understood, several interrelated mechanisms have been proposed, including suppressed bone remodeling via osteoclast inhibition, impaired angiogenesis, soft-tissue toxicity, and microvascular compromise [3].

Within bone tissue, BPs accumulate in hydroxyapatite and are internalized by osteoclasts during bone resorption, where they inhibit key metabolic pathways and induce apoptosis of the osteoclasts [4]. This mechanism undermines the coupling of bone formation and resorption, leading to microdamage accumulation and reduced remodeling capacity [5]. Moreover, impaired angiogenesis and vascular supply have been identified as important co-factors; indeed, denosumab and anti-angiogenic agents also produce MRONJ-like lesions, further implicating vascular dysfunction in the pathogenesis [6]. In this context, secondary inflammation, fibrosis, and oxidative stress within the bone microenvironment further exacerbate necrosis and delay healing [7]. Although no definitive treatment protocol for MRONJ has yet been established, numerous studies have explored a variety of therapeutic approaches aimed at disease control, regression, or symptom alleviation [8–10].

Natural polyphenolic compounds have gained attention for their multi-targeted bioactivities, including antioxidant, anti-inflammatory and bone-modulatory effects [11]. Among them, gallic acid (GA) is a low-molecular-weight phenolic acid found in various plants and fruits, which has been shown to inhibit osteoclastogenesis, suppress pro-inflammatory cytokine expression, and enhance osteoblastic activity in animal models [12]. For instance, Zhang and colleagues (2022) demonstrated that GA significantly inhibited RANKL-induced osteoclast formation and prevented bone loss in an ovariectomized mouse model, likely by blocking protein kinase B, extracellular signal–regulated kinase and c-jun N-terminal kinase pathways [13]. In another study, GA improved bone regeneration in critical-sized calvarial defects in rats, reducing inflammation and osteoclast counts while increasing osteoprotegerin and bone morphogenetic protein-2 (BMP-2) expression [14].

**Fig. 1:**
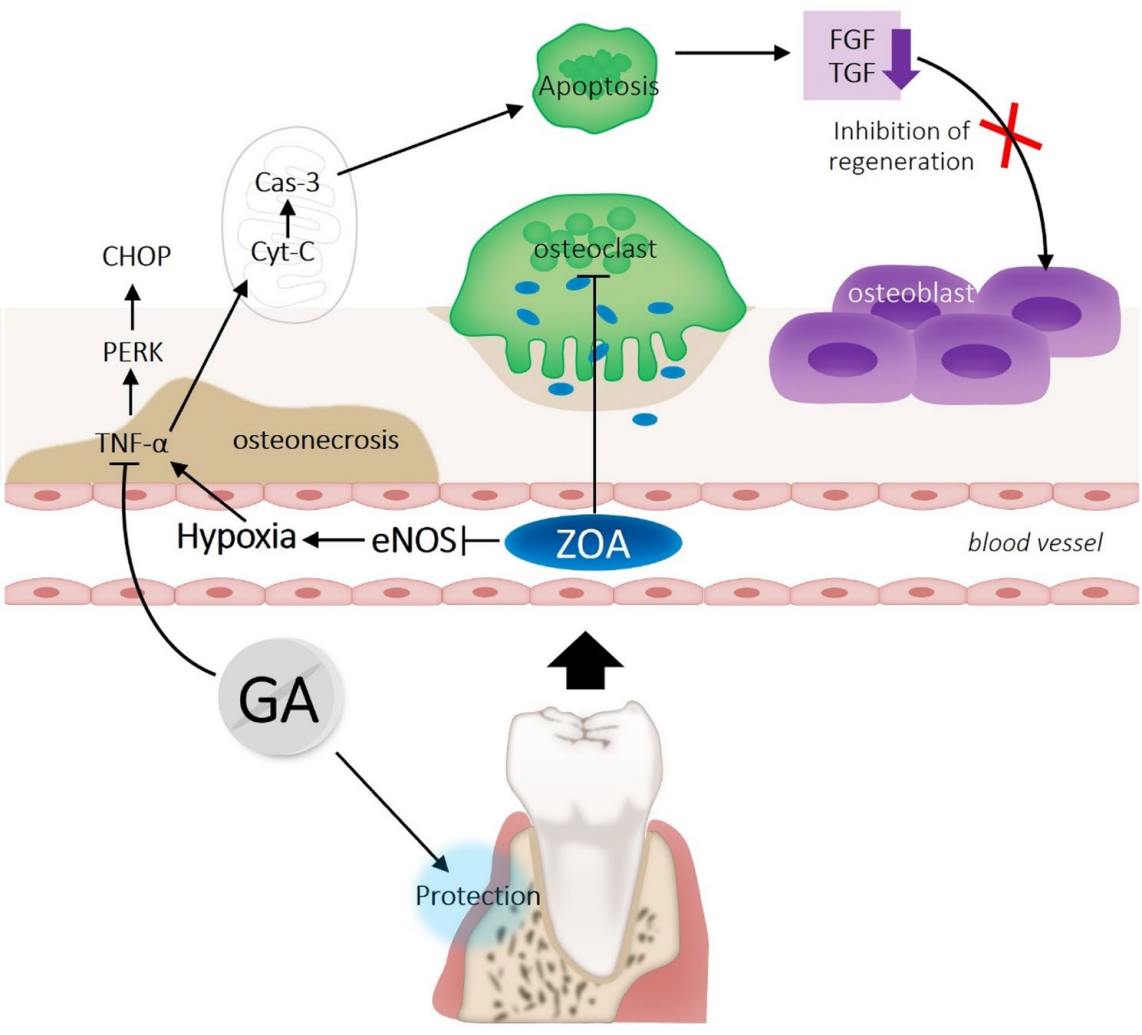
Schematic representation of ZOA-induced osteonecrosis and the potential protective role of GA. ZOA impairs eNOS activity, promotes hypoxia, inflammation (TNF-α), ER stress (PERK–CHOP), and mitochondrial apoptosis (Cyt-C, Cas-3), leading to disrupted bone remodeling. GA mitigates inflammation, oxidative/ER stress, and apoptosis, supporting vascular integrity and balanced osteoblast–osteoclast activity. Cyt-C: Cytochrome c, Cas-3: Caspase-3, ER: Endoplasmic reticulum, FGF: Fibroblast growth factor, TGF: Transforming growth factor, PERK: Protein kinase RNA-Like endoplasmic reticulum kinase, CHOP: C/EBP homologous protein, eNOS: Endothelial nitric oxide synthase, TNF-α: Tumor necrosis factor alpha, ZOA: Zoledronic acid, GA: Gallic acid

Despite these promising data on bone regeneration and remodeling, the specific application of GA in the context of MRONJ has not been comprehensively explored. Given the overlap of mechanisms, excessive osteoclastic suppression, inflammation, fibrosis, oxidative and endoplasmic reticulum (ER) stress in MRONJ and the known activity profile of GA, it is plausible to hypothesize that GA may confer both protective and therapeutic benefits in a BP-induced MRONJ model. Figure 1 illustrates the proposed mechanism underlying ZOA-induced osteonecrosis and the potential protective effects of GA.

In this study, the potential therapeutic and prophylactic effects of GA on ZOA–induced MRONJ in rats were investigated. Histopathological findings, along with immunohistochemical analyses (Fibroblast growth factor [FGF], transforming growth factor beta 1 [TGF-1β], tumor necrosis factor alpha [TNF-α]) and gene expression analyses of growth- and inflammation-related markers (TNF-α, endothelial nitric oxide synthase [eNOS]), as well as mitochondrial (cytochrome c [Cyt-C], caspase-3 [Cas-3]) and endoplasmic reticulum (ER) stress markers (protein kinase RNA-like ER kinase [PERK], C/EBP homologous protein [CHOP]), were evaluated as remodeling-related pathways.

## Materials and methods

### Ethical approval

All experimental procedures were performed in accordance with the ARRIVE 2.0 protocol for animal experiments. The experimental protocol was reviewed and approved by the Suleyman Demirel University Animal Experiments Local Ethics Committee (SDU-HADYEK; approval no: 04/490, approval date: 10/04/2025).

### Animals and experimental protocol

A total of 32 adult female Wistar albino rats (weighing 200–250 g) were obtained from the Süleyman Demirel University Experimental Animal Research Center. Female rats were preferentially selected because MRONJ is more frequently reported in women receiving antiresorptive therapy for osteoporosis and malignancy, and previous experimental MRONJ models have predominantly used female rodents to better reflect this clinical background. Nevertheless, the potential influence of sex on disease susceptibility and response to gallic acid cannot be excluded. Additionally, in a study conducted by Iglesias-Carres et al., no significant differences were observed in plasma or peripheral organ concentrations of gallic acid in rats administered a GA-containing extract. Therefore, considering these findings and the epidemiology of MRONJ, the use of only female animals in the study design was deemed sufficient [15].

All animals were maintained under controlled environmental conditions (temperature 22 ± 2 °C, relative humidity 55 ± 5%, 12-h light/dark cycle) with free access to standard pellet chow and water ad libitum. After a one-week acclimatization period, rats were randomly divided into four experimental groups (*n* = 8 per group) as follows:

1. Control group: Rats received intraperitoneal (i.p.) injections of sterile saline. To ensure equal stress in place of drug administration, injections were performed at the same frequency and with identical volumes.
2. MRONJ group (ZOA + tooth extraction): Rats were administered ZOA (Ostezolen®, Recordati İlaç, Türkiye) i.p. at a dose of 0,06 mg/kg/week for 5 weeks. Saline was administered i.p. instead of GA treatment [16].
3. MRONJ + Pre-GA group (Prophylactic GA): Along with ZOA treatment as above, rats received GA (G7384, Sigma-Aldrich, Sweden) i.p. at a dose of 100 mg/kg/day for 7 days before tooth extraction [16, 17].
4. MRONJ + Post-GA group (Therapeutic GA): Following 5 weeks of ZOA treatment and tooth extraction, rats were given GA 100 mg/kg/day i.p. for 7 days postoperatively [16, 17].

At the end of the 5th week, the left mandibular first and second molar teeth of all rats were extracted under general anesthesia induced by ketamine (Keta-Control, Doğa İlaç, Türkiye) (90 mg/kg, i.p.) and xylazine (XylazinBio, Bioveta, Czech Republic) (8–10 mg/kg, i.p.). Depth of anesthesia was monitored by pedal withdrawal and corneal reflexes. Prior to the surgical procedure, the operative area was isolated with sterile drapes. A sterile surgical instrument set was used for the extractions. After anesthesia was achieved, the rats were secured in position using a custom-made retractor. The left posterior mandibular region was exposed. Using a No. 15 scalpel blade, the left mandibular first and second molar teeth were mobilized, and tooth extraction was completed by applying luxation with a curved, fine-tipped hemostat. Following extraction, sterile gauze was applied to achieve hemostasis. All extractions were performed by the same surgeon using the same technique. After tooth extraction, carprofen (Rimadyl, Zoetis, Türkiye) was administered subcutaneously at a dose of 5 mg/kg for 5 days as an analgesic.

Rats were monitored daily for general health, food and water intake, wound healing, and signs of distress throughout the postoperative 4-week period. At the end of the 9th week, animals were deeply anesthetized (ketamine/xylazine) and euthanized via surgical exsanguination from the vena cava inferior. Mandibular bone segments, including extraction sockets and surrounding soft tissues, were carefully dissected. The specimens were divided for histopathological, immunohistochemical, and genetic analyses.

### Histopathological method

Jaw specimens were harvested during necropsy and fixed in 10% neutral buffered formalin. Following fixation, samples were decalcified for two weeks, dehydrated through graded ethanol, and embedded in paraffin. Section (5 μm) were stained with H&E and examined under a light microscope by a pathologist blinded to group allocation. Lesion areas were quantified using the CellSens Life Science Imaging System. Osteoclast and osteoblast counts were evaluated within a 1.23 mm² area at ×400 magnification [18]. Analyses focused on regions of osteonecrosis, ensuring selection of anatomically comparable sites across all specimens for consistency [18–21]. Histopathological grading of osteonecrotic lesions was performed semi-quantitatively based on necrosis extent, inflammatory infiltration, fibrous tissue formation, osteoblast/osteoclast counts, and collagen organization, as summarized in Table 1.

**Table 1:** Histopathological scoring criteria for osteonecrotic lesions

Parameter / Score	0 (None)	1 (Mild)	2 (Moderate)	3 (Severe)
Extent of Necrosis	No necrotic areas; normal bone architecture preserved	Focal necrosis involving < 25% of the field	Multifocal necrosis involving 25–50% of the field	Extensive necrosis involving > 50% of the field; loss of trabecular structure
Inflammatory Cell Infiltration	Absent	Scattered inflammatory cells	Moderate, diffuse infiltration	Dense, widespread infiltration with tissue destruction
Fibrous Tissue Formation (Fibrosis)	Absent	Minimal fibrous tissue around necrotic foci	Moderate fibroblast proliferation and collagen deposition	Dense fibrotic tissue replacing large necrotic areas
Collagen Organization	Normal, well-organized fibers	Slight disorganization	Moderate loss of organization	Severe disruption and fragmentation of collagen fibers
Osteoclast Number	Normal (0–2 / HPF)	Slightly increased (3–5 / HPF)	Moderately increased (6–10 / HPF)	Markedly increased (> 10/HPF); giant multinucleated forms present
Osteoblast Number	Normal osteoblastic lining	Mildly reduced osteoblastic activity	Markedly reduced osteoblasts, discontinuous lining	Absence of osteoblasts along necrotic bone surfaces

*Abbreviations*: *HPF* high-power field (×400)

### Collagen staining

For Masson’s trichrome staining, sections were deparaffinized and rehydrated as in the standard histopathological protocol. Staining was carried out using a commercial kit (Trichrome Stain Kit—Connective Tissue Stain, ab150686; Abcam, Cambridge, UK) following the manufacturer’s instructions, with light green applied as the counterstain. After staining, sections were dehydrated, cleared in xylene, and mounted with Entellan (Merck, Darmstadt, Germany). Slides were air-dried at room temperature for 24 h before microscopic examination.

### Immunohistochemical method

Primary antibodies used for target protein detection included FGF (FGF-2, clone C-2; sc-74412, Santa Cruz Biotechnology, TX, USA), TGF-1β (Anti-TGF beta 1 antibody [TB21] ab190503, Abcam, Cambridge, UK), VEGF (VEGFA, #AF5131; Affinity Biosciences, Canada), and TNF-α (clone RM1005; ab307164, Abcam, Cambridge, UK). The secondary detection system was purchased from Abcam (Cambridge, UK). Primary antibodies were diluted 1:100 and incubated with tissue sections for 60 min at room temperature. After washing, sections were treated with a biotinylated secondary antibody and a streptavidin–ALP conjugate. Visualization was achieved using the Mouse-Specific HRP/DAB (ABC) Detection IHC Kit (ab64259, Abcam), with diaminobenzidine (DAB) as the chromogen. Negative controls were prepared by omitting the primary antibody, and manufacturer-recommended positive controls were used for each marker. All slides were coded and evaluated by a blinded pathologist. For each slide, the analyzed regions corresponded to the extraction socket and adjacent trabecular bone in anatomically comparable areas across all groups. Immunoreactivity was semi-quantitatively scored for staining intensity on a four-point scale (0 = none, 1 = mild, 2 = moderate, 3 = strong). Image analysis and morphometric evaluations were performed using ImageJ (version 1.48; NIH, Bethesda, MD, USA) and CellSens Life Science Imaging Software (Olympus Co., Tokyo, Japan), and image-derived data were statistically analyzed.

### RT-qPCR

Using the manufacturer’s protocol, RNA was isolated from homogenized tissues with the GeneAll RiboEx (TM) RNA Isolation Kit (GeneAll Biotechnology, Seoul, Korea). The amount and purity of the RNAs obtained were measured with the BioSpec-nano nanodrop (Shimadzu Ltd. Kyoto, Japan) device. 1 µg RNA was used for cDNA synthesis. cDNA synthesis, A.B.T. ™ cDNA Synthesis Kit (Atlas Biotechnology, Türkiye) was carried out in a thermal cycler according to the protocol. Primer designs were made by detecting specific mRNA sequences and testing possible primer sequences using the NCBI website. The sequences of the primer sequences used are shown in Table 2. Expression levels of genes were measured in a Biorad CFX96 (California/USA) real-time PCR instrument using 2X SYBR green master mix (Nepenthe/Türkiye). In the study, the ACTB gene was used as a housekeeping gene. The reaction mixture was prepared according to the manufacturer’s protocol to a final volume of 20 µl. The resulting reaction mixture was placed in a real-time qPCR device determined according to the kit manufacturer’s protocol, and each sample was studied in 3 replications. PCR conditions, initial denaturation 94 °C 10 min. 1 cycle, denaturation 95 °C 15 s. and annealing/extension 55 °C 30 s. was applied as 40 cycles. Relative mRNA levels were calculated by applying the 2^− ΔΔCt^ formula to the normalized results.

**Table 2:** Primary sequences, product size and accession numbers of genes

Genes	Primary sequence	Product size	Accession number
ACTB (HouseKeeping)	F: CCCGCGAGTACAACCTTCTT	481 bp	NM_031144.3
	R: AACACAGCCTGGATGGCTAC		
TNF-α	F: TCGTAGCAAACCACCAAGCA	208 bp	NM_012675.3
	R: GAAGTGGCAAATCGGCTGAC		
TGF-1β	F: CCAGATCCTGTCCAAACTAA	218 bp	NM_021578.2
	R: TTTTGTCATAGATTGCGTTG		
eNOS	F: GGTTGACCAAGGCAAACCAC	247 bp	NM_021838.2
	R: CCTAATACCACAGCCGGAGG		
CHOP	F: TGGAAGCCTGGTATGAGGATCTG	175 bp	XM_006241445.4
	R: GAGGTGCTTGTGACCTCTGCTG		
PERK	F: CCAAGCTGTACATGAGCCCAGA	178 bp	XM_008762977.4
	R: TTCTGAGTGAACAGTGGTGGAAAC		
Cyt-C	F: TAAATATGAGGGTGTCGC	192 bp	NM_012839.2
	R: AAGAATAGTTCCGTCCTG		
Cas-3	F: GGCCGACTTCCTGTATGCTT	110 bp	NM_001436899.1
	R: CGTACAGTTTCAGCATGGCG		

*F* Forward, *R *Reverse, *ACTB *Actin B, *TNF-α *Tumor necrosis factor, *TGF-1β *Transforming Growth Factor Beta 1, *eNOS *Endothelial nitric oxide synthase, *CHOP *C/EBP homologous protein, *PERK* Eukaryotic translation initiation factor 2 alpha kinase 3, *Cyt-C *Cytochrome C, *Cas-3 *Caspase 3

### Statistical analyses

For statistical analysis, histopathological, immunohistochemical and genetic scores were compared between the groups. For this purpose, the One-way ANOVA with Tukey’s multiple comparison tests was used by the GraphPad Prism version 10.1 (GraphPad Software, Boston, MA, USA) package program. The level of significance was considered at *p* < 0.05.

## Results

### Histopathology

Histopathological evaluation revealed marked degenerative alterations in the MRONJ group, including extensive necrotic areas, inflammatory infiltration, and fibrotic replacement of bone trabeculae. Semi-quantitative scoring demonstrated a significant increase in necrosis scores in the MRONJ group compared with the control group (*p* < 0.001). Both GA regimens significantly lowered necrosis scores (*p* < 0.001 vs. MRONJ), indicating a partial preservation of bone viability rather than complete normalization.

Inflammatory scores were also significantly elevated in the MRONJ group (*p* < 0.01), reflecting dense infiltration of neutrophils and macrophages around necrotic bone surfaces. GA administration reduced inflammation, particularly in the pre-treatment group (*p* < 0.05 vs. MRONJ), although low-grade inflammatory foci were still detectable in some specimens.

Fibrosis analysis showed abundant fibroblast proliferation and excessive collagen deposition replacing necrotic bone in the MRONJ group (*p* < 0.001 vs. Control). Both GA regimens markedly prevented fibrotic tissue accumulation, with a more organized connective-tissue pattern in GA-treated specimens (*p* < 0.001 vs. MRONJ).

Collagen organization scores, as quantified by Masson’s trichrome staining, revealed severe disorganization and fragmentation in the MRONJ group (*p* < 0.001). GA pre-treatment significantly improved collagen fiber alignment (*p* < 0.001), whereas the post-treatment group displayed moderate restoration (*p* < 0.05).

**Fig. 2:**
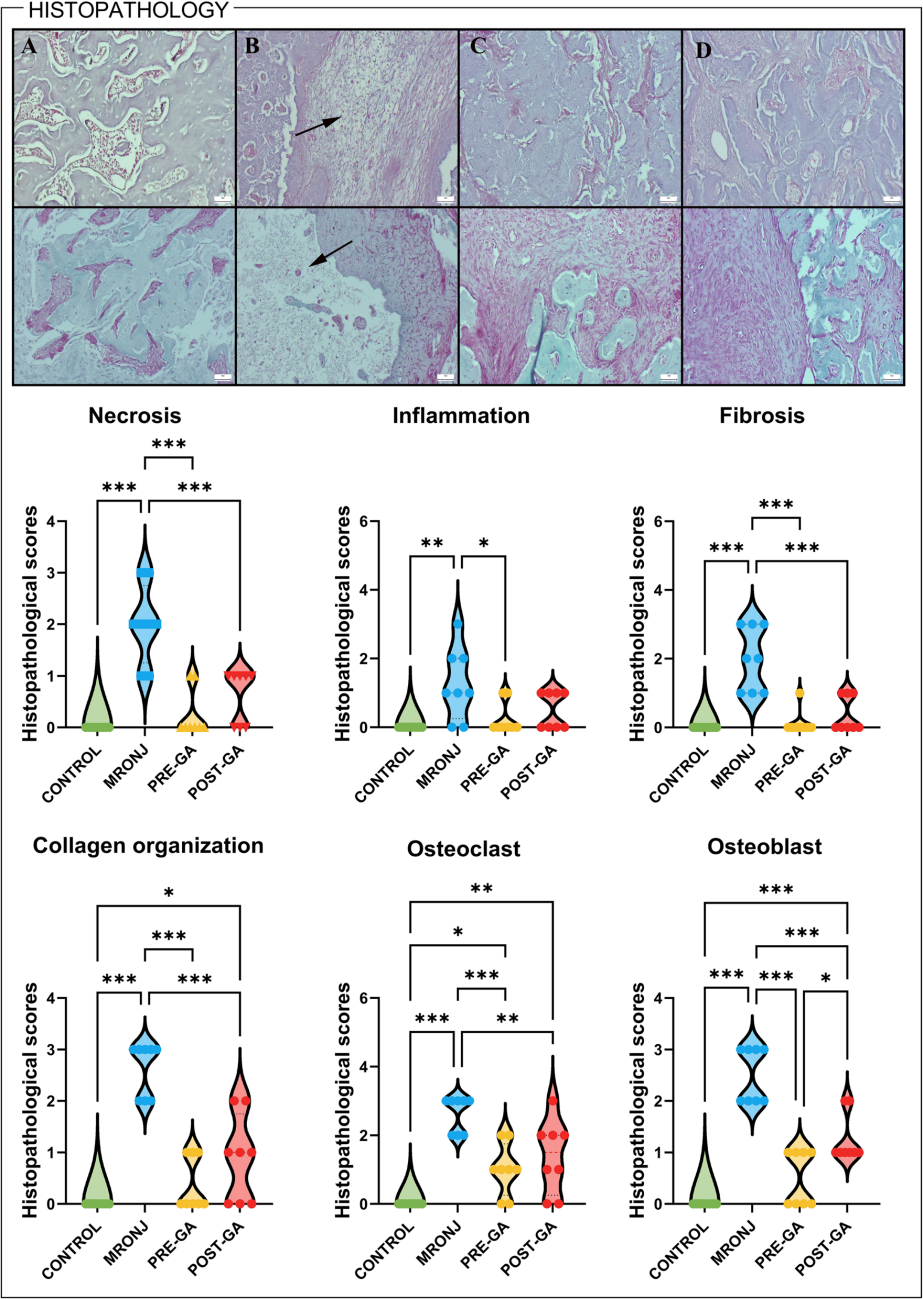
Histopathological (upper row) and collagen (lower row) analysis of mandibular bone tissues across experimental groups. **A** Control group: Normal bone histoarchitecture with well-organized lamellar structure and intense red collagen staining, indicating preserved matrix integrity. **B** MRONJ group: Extensive osteonecrotic regions (arrow) characterized by empty areas and disrupted lamellar architecture. **C** MRONJ + Pre-GA group: Marked histological improvement with restoration of bone organization, reduced osteoclastic activity, minimal necrotic areas, and dense, well-aligned collagen fibers, suggesting a strong protective effect of GA pre-treatment. **D** MRONJ + Post-GA group: Minimal residual necrotic foci and nearly normal connective tissue structure, with moderate collagen intensity and organization comparable to healthy bone. Hematoxylin and eosin staining (upper row) and Masson’s trichrome staining (lower row); scale bars = 50 μm. Violin plots depict semi-quantitative histopathological scores for necrosis, inflammation, fibrosis, collagen organization, osteoclast count, and osteoblast activity. Data were analyzed using one-way ANOVA with Tukey’s post hoc test (*p < 0.05, **p < 0.01, **p < 0.001). MRONJ: Medication-related osteonecrosis of the jaw, GA: Gallic acid

Osteoclast counts were markedly elevated in MRONJ animals (*p* < 0.001), with numerous multinucleated giant cells showing vacuolization and irregular morphology. Both GA-treated groups exhibited significantly reduced osteoclast activity (*p* < 0.01 for post-GA; *p* < 0.001 for pre-GA). Osteoblast scores were also significantly increased in the MRONJ group (*p* < 0.001), reflecting a pronounced reduction in osteoblastic lining according to the scoring system; GA treatment decreased these scores and partially restored the continuity of osteoblastic coverage along viable bone surfaces (*p* < 0.001 vs. MRONJ) (Fig. 2).

### Immunohistochemistry

Immunohistochemical analyses demonstrated marked alterations in the expression of regulatory markers associated with osteogenesis and inflammation across experimental groups.

In the MRONJ group, FGF immunoreactivity was significantly reduced compared with the Control group (*p* < 0.05). Both GA pre- and post-treatment tended to increase FGF staining intensity toward control levels, although these changes did not reach statistical significance.

Similarly, TGF-1β expression was markedly downregulated in the MRONJ group (*p* < 0.001 vs. Control). Administration of GA improved TGF-1β immunoexpression, but no significant differences were detected between MRONJ and GA-treated groups based on semi-quantitative scoring.

**Fig. 3:**
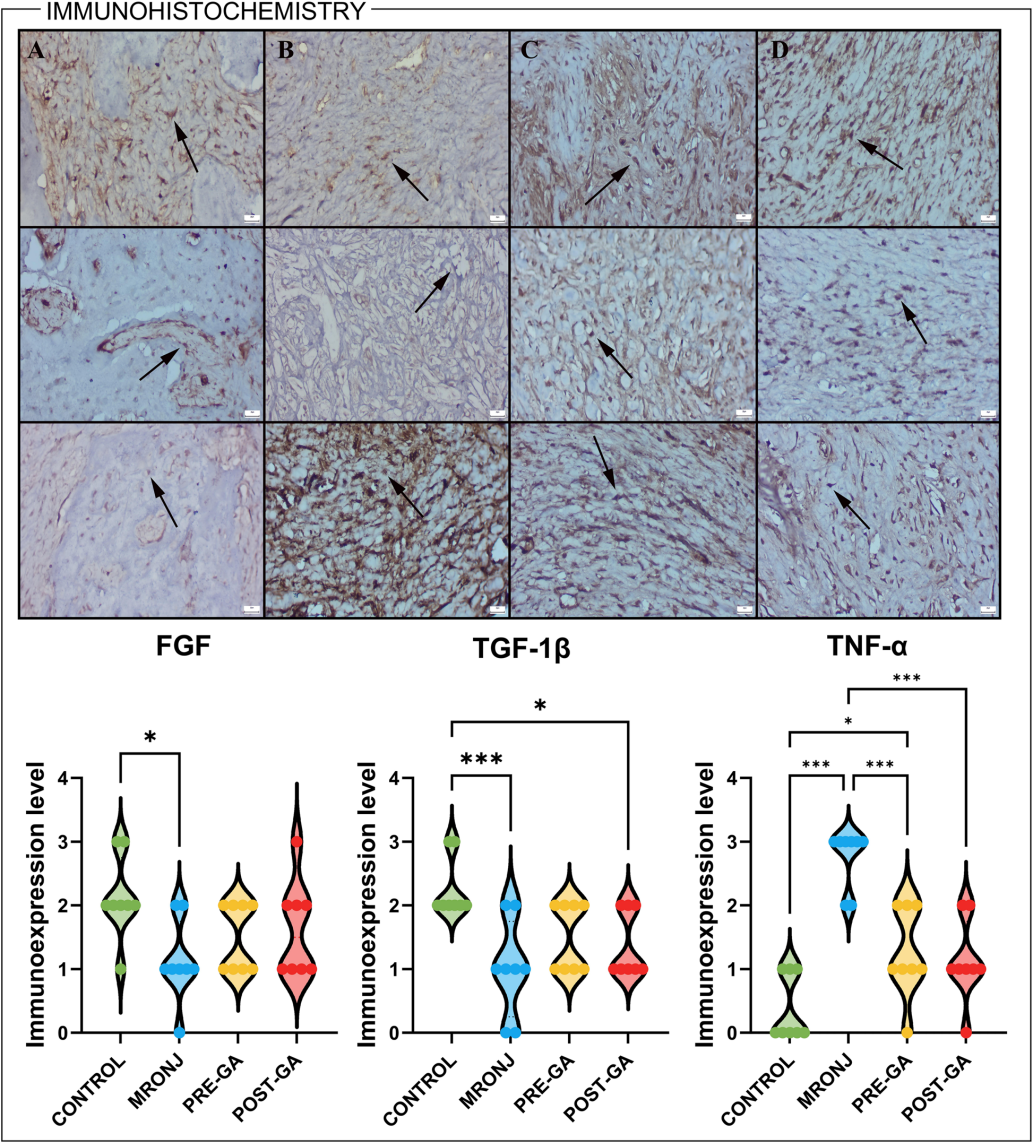
Immunohistochemical expression profiles of FGF (upper row), TGF-1β (middle row) and TNF-α (lower row) across experimental groups. **A** Control group: Normal basal expression of all markers, reflecting balanced osteogenic and inflammatory signaling. **B** MRONJ group: Markedly reduced FGF and TGF immunoreactivity with strong TNF-α expression, indicative of suppressed reparative activity and enhanced inflammation within osteonecrotic areas. **C** MRONJ + Pre-GA group: Pronounced restoration of FGF and TGF expression coupled with a distinct reduction in TNF-α staining intensity, suggesting effective modulation of inflammation and promotion of tissue regeneration. (**D** MRONJ + Post-GA group: Elevated FGF and TGF expression and decreased TNF-α immunoreactivity, consistent with partial recovery of bone remodeling dynamics. Arrows indicate immunopositive cells. Streptavidin–biotin peroxidase method; scale bars = 20 μm. Data were analyzed using one-way ANOVA with Tukey’s post hoc test (*p < 0.05, *** *p* < 0.001). MRONJ: Medication-related osteonecrosis of the jaw, GA: Gallic acid, FGF: Fibroblast growth factor, TGF-β: Transforming Growth Factor Beta, TNF-α: Tumor necrosis factor alpha

Conversely, TNF-α immunoreactivity was dramatically elevated in the MRONJ specimens (*p* < 0.001). Both GA treatment regimens significantly reduced TNF-α levels (*p* < 0.001 vs. MRONJ for both) (Fig. 3).

### Gene expression results

#### Inflammation, fibrosis and vascularization gene profiles

Quantitative RT-PCR analyses revealed significant alterations in the expression of inflammatory, fibrotic, and vascular markers in MRONJ-affected mandibular tissues.

TNF-α mRNA levels were markedly elevated in the MRONJ group compared with controls (*p* < 0.001). Both GA pre- and post-treatment significantly downregulated TNF-α expression (*p* < 0.01 and *p* < 0.01, respectively), with pre-treatment achieving a greater reduction.

Similarly, TGF-1β mRNA expression was significantly upregulated in the MRONJ group (*p* < 0.001), reflecting excessive fibroblast activation and pathological collagen deposition. In the both GA treatment groups TGF-1β transcription levels effectively normalized (*p* < 0.001 for both), with the pre-treatment group exhibiting near-baseline expression.

**Fig. 4:**
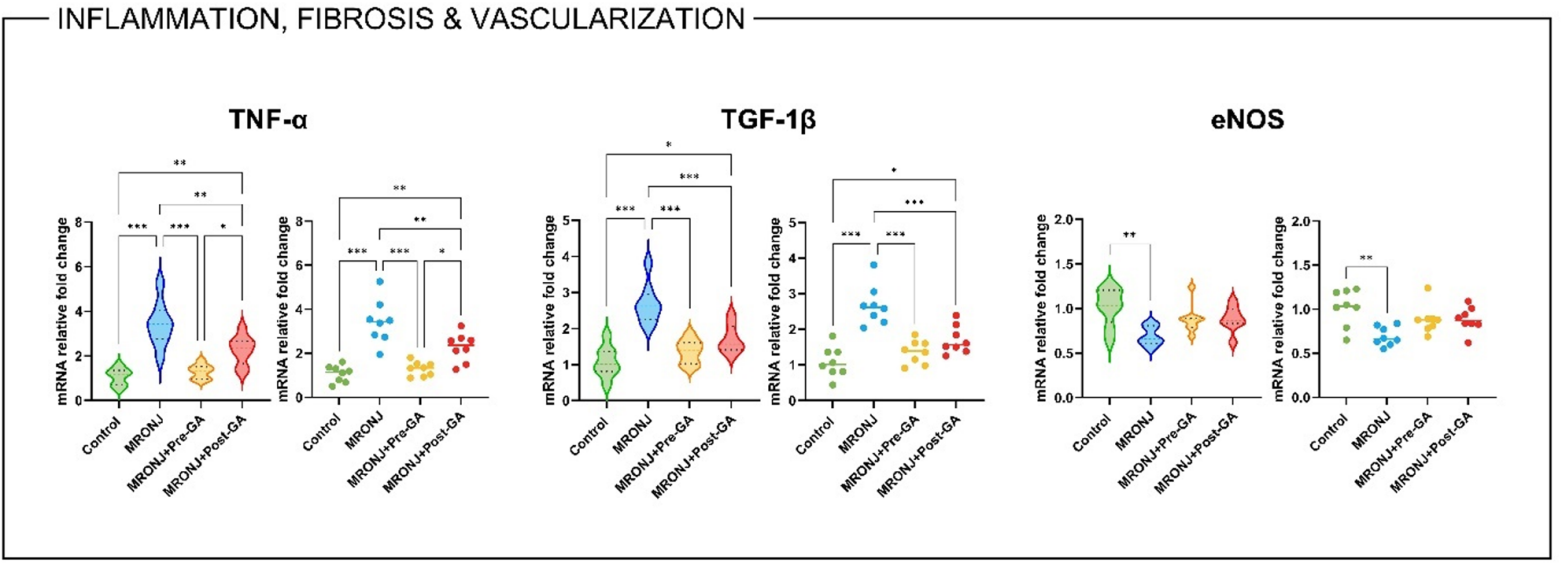
Relative mRNA expression levels of inflammatory, fibrotic, and vascular markers across experimental groups. Bar plots show mean ± SD fold-changes in gene expression. Data were analyzed using one-way ANOVA with Tukey’s post hoc test (*p < 0.05, **p < 0.01, **p < 0.001). MRONJ: Medication-related osteonecrosis of the jaw, GA: Gallic acid, TNF-α: Tumor necrosis factor alpha, eNOS: Endothelial nitric oxide synthase, TGF-1β: Transforming growth factor 1 beta

In contrast, eNOS mRNA expression, a key endothelial and vascularization marker, was significantly reduced in the MRONJ group (*p* < 0.01 vs. Control). Both GA regimens partially restored eNOS expression, although the change was not significant (Fig. 4).

#### Mitochondrial stress and apoptosis

**Fig. 5:**
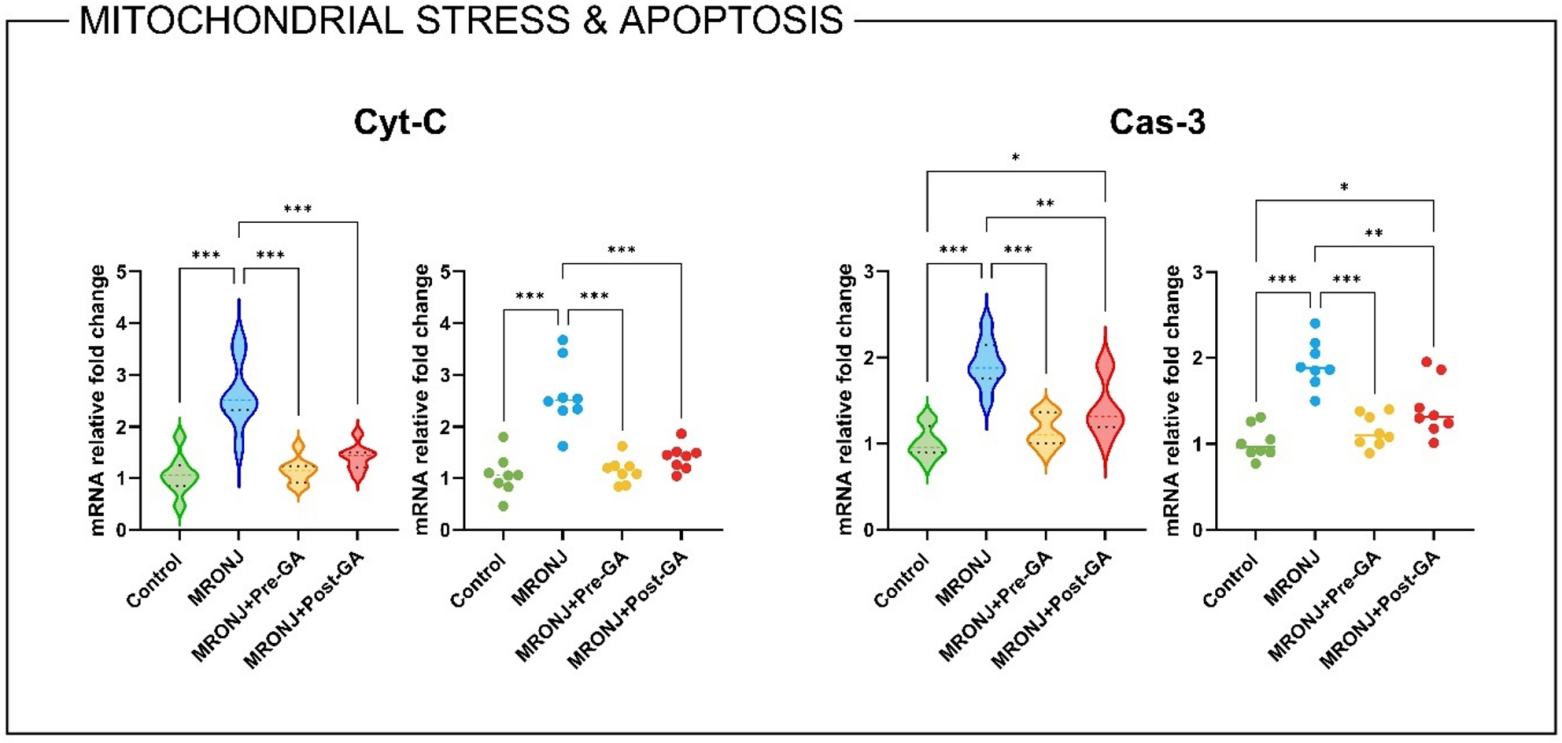
Relative mRNA expression of mitochondrial stress and apoptotic markers. Bar plots show mean ± SD fold-changes in gene expression. Data were analyzed using one-way ANOVA with Tukey’s post hoc test (*p < 0.05, **p < 0.01, **p < 0.001). MRONJ: Medication-related osteonecrosis of the jaw, GA: Gallic acid, Cyt-C: Cytochrome c, Cas-3: Caspase-3

Gene expression analysis revealed that mitochondrial dysfunction and apoptosis were markedly activated in the MRONJ group (Fig. 5). Cyt-C mRNA expression was significantly upregulated in the MRONJ group compared with the control (*p* < 0.001). Both GA pre- and post-treatments effectively suppressed Cyt-C expression (*p* < 0.001 and *p* < 0.01, respectively). The pre-treatment regimen showed near-normal transcript levels, implying a preventive advantage over the post-treatment group.

Similarly, Cas-3 expression—an executioner caspase directly downstream of Cyt-C release—was markedly increased in the MRONJ animals (*p* < 0.001 vs. Control). GA treatment significantly reduced Cas-3 expression (*p* < 0.001 for pre-treatment; *p* < 0.01 for post-treatment) (Fig. 5).

#### ER stress

To further elucidate the molecular mechanisms underlying osteonecrosis and the protective role of GA, ER stress–related gene expression was evaluated. The CHOP transcript, a hallmark pro-apoptotic marker of unresolved ER stress, was significantly upregulated in the MRONJ group compared with the control (*p* < 0.001). Both GA pre- and post-treatment markedly suppressed CHOP mRNA expression (*p* < 0.001 vs. MRONJ for both), with pre-treatment achieving near-normal levels.

**Fig. 6:**
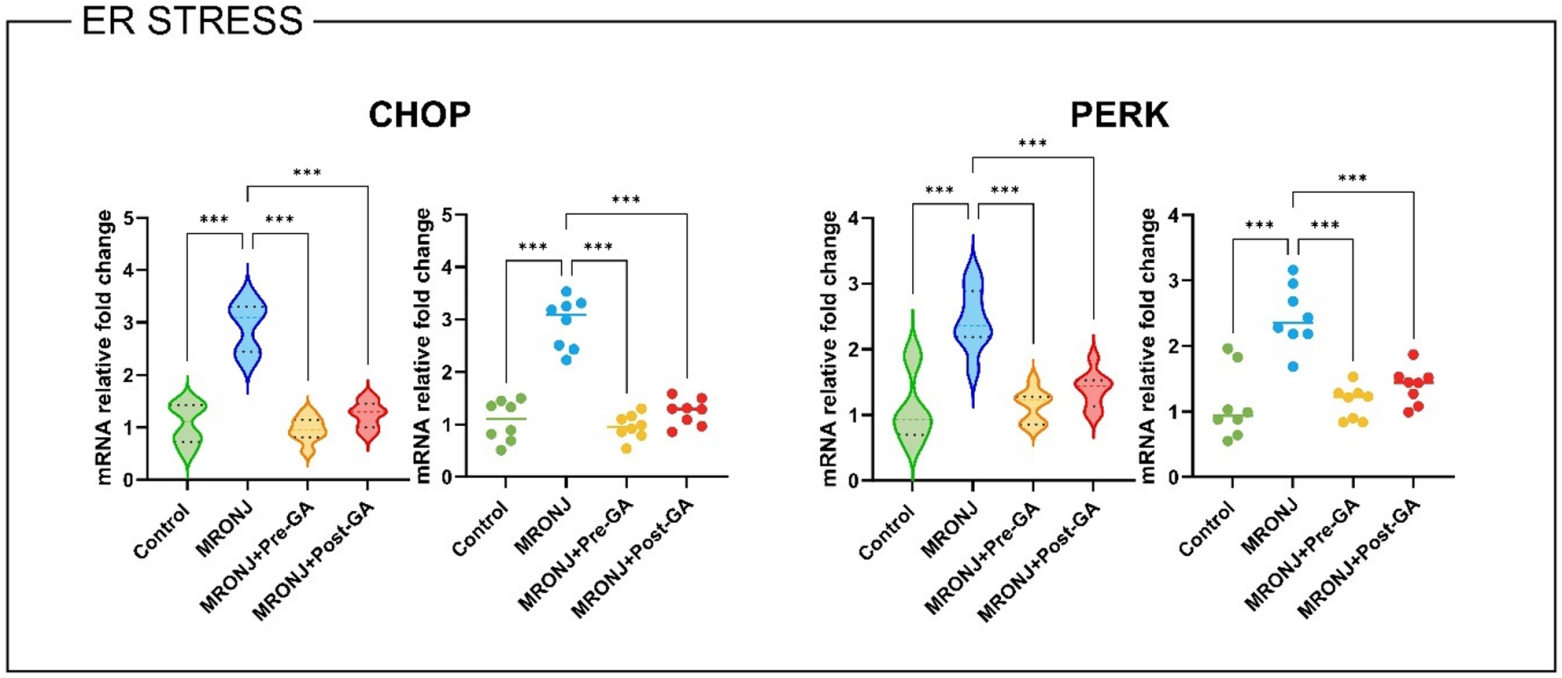
Relative mRNA expression of ER stress markers across experimental groups. Bar plots show mean ± SD fold-changes in gene expression. Data were analyzed using one-way ANOVA with Tukey’s post hoc test (*p < 0.05, **p < 0.01, **p < 0.001). MRONJ: Medication-related osteonecrosis of the jaw, GA: Gallic acid, CHOP: C/EBP homologous protein, PERK: Protein kinase RNA-Like endoplasmic reticulum kinase

Similarly, PERK, an upstream stress-sensor kinase, exhibited a robust increase in mRNA levels in MRONJ specimens compared to the control group (*p* < 0.001). Both GA administrations significantly reduced PERK expression (*p* < 0.001), again more prominently in the pre-treatment group (Fig. 6).

Table 3 presents a comprehensive statistical summary of the histopathological, immunohistochemical, and molecular parameters. Intergroup analyses identified distinctive pathological and biochemical signatures characterizing ZOA-induced MRONJ and underscoring the multifactorial protective role of GA.

**Table 3:** Summary of *p*-values for intergroup comparisons

Parameter	Control vs. MRONJ	Control vs. PRE	Control vs. POST	MRONJ vs. PRE	MRONJ vs. POST	PRE vs. POST
**Histopathology**						
Necrosis	< 0.001	= 0.765	= 0.093	< 0.001	< 0.001	= 0.473
Inflammation	= 0.002	= 0.855	= 0.397	= 0.017	= 0.102	= 0.855
Fibrosis	< 0.001	= 0.970	= 0.545	< 0.001	< 0.001	= 0.808
Collagen organization	< 0.001	= 0.539	= 0.019	< 0.001	< 0.001	= 0.294
Osteoclast count	< 0.001	= 0.038	= 0.003	< 0.001	= 0.007	= 0.710
Osteoblast count	< 0.001	= 0.133	< 0.001	< 0.001	< 0.001	= 0.110
**Immunohistochemistry**						
FGF	= 0.021	= 0.235	= 0.421	= 0.654	= 0.421	= 0.980
TGF-1β	< 0.001	= 0.067	= 0.0265	< 0.329	< 0.573	= 0.972
TNF-α	< 0.001	= 0.029	= 0.075	< 0.001	< 0.001	= 0.974
**RT-qPCR**						
CHOP	< 0.001	= 0.923	= 0.764	< 0.001	< 0.001	= 0.394
PERK	< 0.001	= 0.996	= 0.451	< 0.001	< 0.001	= 0.587
Cyt-C	< 0.001	= 0.981	= 0.380	< 0.001	< 0.001	= 0.601
Cas-3	< 0.001	= 0.705	= 0.019	< 0.001	= 0.002	= 0.188
eNOS	= 0.01	= 0.322	= 0.330	= 0.096	= 0.093	> 0.999
TGF-1β	< 0.001	= 0.566	= 0.035	< 0.001	< 0.001	= 0.403
TNF-α	< 0.001	= 0.897	= 0.005	< 0.001	= 0.006	= 0.02

One-way ANOVA followed by Tukey’s post hoc test was used for statistical comparisons. *MRONJ *Medication-related osteonecrosis of the jaw, *GA *Gallic acid, *Cyt-C *Cytochrome c, *Cas-3 *Caspase-3, *FGF *Fibroblast growth factor, *TGF *Transforming Growth Factor, *PERK *Protein kinase RNA-Like endoplasmic reticulum kinase, *CHOP *C/EBP Homologous Protein, *eNOS* Endothelial nitric oxide synthase

## Discussion

The present study demonstrated that both prophylactic and therapeutic administration of GA markedly ameliorated ZOA–induced MRONJ in rats through the attenuation of inflammation, fibrosis, and stress-mediated apoptosis. Histopathological evaluation revealed extensive necrotic bone areas, inflammatory cell infiltration, and disrupted collagen organization in the MRONJ group, consistent with previous reports that BP exposure leads to suppression of bone remodeling and defective vascularization [1, 22]. In contrast, GA treatment restored trabecular integrity, decreased osteoclastic hyperactivity, and preserved osteoblastic lining continuity—findings comparable with those reported by Altan et al. (2020), who observed improved calvarial bone healing with GA-loaded liposomes [14].

Inflammatory infiltration, a defining feature of MRONJ lesions, reflects activation of toll-like receptor and NF-κB signaling pathways that amplify TNF-α and IL-1β production [23]. In our study, TNF-α immunoreactivity and mRNA expression were significantly elevated in the MRONJ group, indicating a strong pro-inflammatory environment [24]. This observation is consistent with the findings of Lim et al. (2010), who demonstrated that intravenous BP administration increased TNF-α and IL-6 levels, contributing to acute inflammatory activation [25]. GA administration reversed these changes, markedly downregulating TNF-α both at protein and transcriptional levels. These anti-inflammatory effects of GA are supported by Zhang et al. (2022), who demonstrated that GA inhibits NF-κB translocation and cytokine release in RANKL-stimulated osteoclasts [13]. The robust suppression of TNF-α observed here may thus reflect GA’s ability to neutralize ROS-driven signaling and maintain macrophage polarization toward a reparative phenotype.

Histologically, MRONJ lesions are characterized by disorganized collagen bundles and excessive fibrotic deposition, as confirmed by our Masson’s trichrome staining. The severe fibrosis observed in the MRONJ group likely represents a compensatory wound-healing response to chronic inflammation and necrosis [26]. GA-treated rats showed significantly reduced fibrotic tissue accumulation and improved collagen orientation, suggesting inhibition of TGF-β1–driven myofibroblast activation. Supporting this, Ryu et al. (2016) demonstrated that gallic acid reduces Smad3 phosphorylation and collagen type I expression in fibrotic models [27]. This is consistent with our histopathological data demonstrating dense but well-organized collagen networks in GA groups, indicating a shift from pathological scarring toward regenerative remodeling.

Interestingly, our study revealed a discrepancy between TGF-1β immunohistochemical expression and its mRNA levels. While TGF-1β staining was markedly decreased in MRONJ tissues and restored after GA treatment, qPCR showed elevated TGF-1β transcripts in the MRONJ group. This paradox can be explained by the context-dependent duality of TGF-1β signaling. Under chronic inflammatory or fibrotic stress, TGF-1β transcription is upregulated as a compensatory repair signal, but post-translational activation and receptor signaling may be inhibited by oxidative stress, ER stress, or excessive BP accumulation [28, 29]. Thus, despite higher mRNA levels, the bioactive protein fraction and downstream signaling (e.g., p-Smad2/3) are often suppressed in MRONJ lesions [30]. GA appears to restore functional TGF-1β signaling by reducing ROS-mediated inactivation, thereby normalizing protein expression and re-establishing controlled matrix synthesis.

The MRONJ group displayed increased osteoclast counts with giant multinucleated forms, suggesting excessive but ineffective bone resorption. Similar findings were described by Gross et al. (2017) and Kızıldağ et al. (2025), who observed distorted osteoclast morphology in BP -treated animals [21, 31]. GA pre-treatment markedly normalized osteoclast number and morphology, potentially via inhibition of RANKL-induced NF-κB and MAPK cascades. Concomitantly, the osteoblastic population, which was severely depleted in the MRONJ group, was restored by GA administration. Previous work by Zhang et al. (2022) and Altan et al. (2020) similarly showed that GA enhances osteoblast differentiation by activating Runt-related transcription factor-2 and BMP-2 expression [13, 14]. Together, these data indicate that GA reinstates the osteoclast–osteoblast coupling necessary for balanced bone turnover.

Vascular compromise is another critical driver of MRONJ pathogenesis [32]. We found that eNOS gene expression was significantly suppressed in the MRONJ group, consistent with endothelial dysfunction and reduced nitric-oxide availability. GA treatment partially restored eNOS levels, indicating improved angiogenic and vasodilatory capacity. These findings mirror those of Hadidi et al. (2024), who demonstrated that GA upregulates eNOS and VEGF in ischemic tissues by enhancing nuclear factor erythroid 2-related factor 2 Heme oxygenase-1 signaling [33]. The partial, rather than complete, restoration observed here likely reflects the irreversible microvascular damage caused by prolonged BP exposure, emphasizing the benefit of prophylactic GA administration before osteonecrosis establishment.

Our qPCR analysis revealed a marked upregulation of CHOP, PERK, Cyt-C, and Cas-3 transcripts in the MRONJ group, indicating concurrent ER and mitochondrial stress, consistent with the findings of Fan et al. (2021), who demonstrated oxidative stress–induced mitochondrial dysfunction and caspase-dependent osteocyte apoptosis in steroid-induced bone necrosis [34]. As is well established, CHOP activation has been associated with disruption of the balance between pro-apoptotic and anti-apoptotic proteins at the mitochondrial membrane, thereby promoting the release of Cyt-C into the cytosol. Therefore, the co-activation of PERK/CHOP and Cyt-C/Cas-3 pathways may indicate crosstalk between ER stress and intrinsic apoptosis cascades [35]. GA significantly suppressed both pathways, which aligns with its known capacity to modulate unfolded protein response and to scavenge free radicals that destabilize mitochondrial membranes [36]. These findings suggest that GA acts as a mitochondrial-protective and ER-stabilizing compound, reducing cellular apoptosis and preserving osteocyte viability.

Taken together, the present results support a multifaceted mechanism whereby GA exerts anti-inflammatory, anti-fibrotic, and anti-apoptotic effects in MRONJ. By concurrently downregulating TNF-α and CHOP while restoring TGF-β and eNOS, GA appears to re-establish a microenvironment conducive to tissue regeneration. This integrative regulation of oxidative, inflammatory, and stress pathways underscores GA’s potential as a pharmacological adjunct in patients at high risk for MRONJ. Moreover, prophylactic administration provided greater histological and molecular protection than post-treatment, implying that timing of antioxidant therapy is crucial for efficacy.

This study has several limitations that should be considered when interpreting the findings. First, osteoclast activity was assessed by histomorphometric counting on H&E-stained sections rather than TRAP or other osteoclast-specific histochemical staining. While our scoring system captured relative changes in osteoclast number and morphology, the lack of TRAP staining limits the precision of conclusions regarding osteoclast function. Second, we investigated prophylactic and therapeutic regimens separately to distinguish preventive from reparative effects, but a combined pre- and post-treatment protocol was not evaluated and may yield different outcomes. Third, mechanistic interpretations regarding ER stress and mitochondrial apoptosis are based solely on in vivo gene-expression data. No complementary in vitro experiments were performed to dissect upstream signaling events or cell-type–specific responses, which should therefore be regarded as hypothesis-generating rather than definitive. Finally, although GA demonstrated substantial efficacy in rats, clinical translation will require pharmacokinetic evaluation and optimization of delivery systems to overcome oral bioavailability constraints [37]. Future work should also explore the combination of GA with angiogenic or osteoinductive biomaterials, directly assess osteoclast function with TRAP staining, and incorporate in vitro models to validate the proposed molecular pathways and evaluate long-term effects on bone regeneration.

In conclusion, our findings support the hypothesis that GA can attenuate ZOA–induced osteonecrosis by suppressing inflammatory cytokines, reducing fibrotic remodeling, restoring osteoblast function, and modulating ER–mitochondrial stress. Given the preclinical nature of the model and the limitations outlined above, these results should be interpreted as preliminary but encouraging. Together, they highlight the potential of GA as a biologically safe, naturally derived adjunct that may contribute to the prevention and management of MRONJ in high-risk patients, warranting further mechanistic and clinical investigations.

## Data Availability

The datasets used and/or analyzed during the current study are available from the corresponding author on reasonable request.

## Abbreviations

BP: Bisphosphonate
BMP-2: Bone morphogenetic protein-2
BUN: Blood urea nitrogen
Cas-3: Caspase-3
CHOP: C/EBP homologous protein
Cyt-C: Cytochrome-C
DAB: 3,3′-Diaminobenzidine
ER: Endoplasmic reticulum
eNOS: Endothelial nitric oxide synthase
FGF: Fibroblast growth factor
GA: Gallic acid
H&E: Hematoxylin and eosin
HPF: High-power field
IHC: Immunohistochemistry
IL-1β: Interleukin-1 Beta
IL-6: Interleukin-6
MAPK: Mitogen-activated protein kinase
MRONJ: Medication-related osteonecrosis of the jaw
NF-κB: Nuclear factor kappa-light-chain-enhancer of activated B cells
PBS: Phosphate buffered saline
PERK: Protein kinase RNA-like endoplasmic reticulum kinase
RT-qPCR: Reverse transcription-polymerase chain reaction
RANKL: Receptor activator of nuclear factor kappa-B ligand
ROS: Reactive oxygen species
SD: Standard deviation
TAS: Total Antioxidant status
TGF-1β: Transforming growth factor 1 beta
TNF-α: Tumor necrosis factor alpha
TOS: Total Oxidant Status
VEGF: Vascular endothelial growth factor
ZOA: Zoledronic acid
